# Short-Term Cold Stress Affects Parasitism on the Asian Chestnut Gall Wasp *Dryocosmus kuriphilus*

**DOI:** 10.3390/insects11120841

**Published:** 2020-11-28

**Authors:** Carmelo Peter Bonsignore, Giusi Vizzari, Gregorio Vono, Umberto Bernardo

**Affiliations:** 1 Laboratorio di Entomologia ed Ecologia Applicata, Dipartimento PAU, Università Mediterranea di Reggio Calabria, 89124 Reggio Calabria, Italy; vizzarigiusi@gmail.com; 2 Dipartimento di Agraria, Università degli Studi Mediterranea di Reggio Calabria, 89122 Reggio Calabria, Italy; gregorio.vono@unirc.it; 3 CNR, Institute for Sustainable Plant Protection, SS of Portici, 80055 Portici, Italy; umberto.bernardo@ipsp.cnr.it

**Keywords:** chestnut, climate, thermal variation, Cynipidae, *Dryocosmus kuriphilus*, *Eupelmus* spp., fitness, host–parasitoid relationship

## Abstract

**Simple Summary:**

The Asian cynipid gall wasp (ACGW) “*Dryocosmus kuriphilus*” has become widespread in Europe. In all invaded areas, it is parasitized by native parasitoids associated with oak galls, for which the ACGW represents a non-saturated adaptation space. Considering the increase in the frequency of extreme climatic events over the last twenty years (e.g., low temperatures during the vegetative period of the chestnut tree), this study aimed to elucidate the effects of cold stress on both ACGW biology and parasitism by native and introduced parasitoids. The ACGW–parasitoid system represents an ideal subject in which to evaluate the effect of sudden cold stress events due to the wasps’ biological characteristics, which include the ability to complete development even in galls detached from plants. We show that parasitism on and the mortality of ACGWs in three chestnut fields were affected by a cold treatment. Our results reveal species-specific differences in the abundance and performance of parasitoids associated with the ACGW in response to cold stress. For example, the frequency of *Eupelmus* spp. and *Mesopolobus tibialis* doubled as a result of the cold treatment in all three chestnut fields in both study years. Therefore, the plasticity in response to short-term temperature variation is associated with individual fitness in some parasitoid species.

**Abstract:**

Temperature variation affects interactions involving plants, herbivores, and parasitoids, causing a mismatch between their phenological cycles. In the context of climate change, climatic factors can undergo profound and sudden changes, such as sudden hot or cold snaps. Herein, we show that the number of episodes of short but sustained low temperatures has increased, mainly during May, over the last two decades. We subjected galls induced by the Asian chestnut gall wasp (ACGW) *Dryocosmus kuriphilus* to cold stress to assess whether and, if so, how it affected the pest and its parasitoids. Over the course of two years, we measured seasonal parasitism, parasitism rates, the relative abundance of each parasitoid species, and ACGW mortality. We found that the cold treatment affected both the pest and the parasitoids, resulting in a reduction in the emergence of ACGWs and differing ratios of species within the parasitoid community. The most striking example was the change in the relative frequency of three species of *Eupelmus* spp. and *Mesopolobus tibialis*, which doubled in cold-stressed galls in all chestnut fields. The effects of temperature on the development of the host and the direct effects of cold temperatures on the surface of galls (in terms of the humidity or hardness of the galls) warrant further research in this direction.

## 1. Introduction

In the context of global climate change, climatic factors can undergo profound and sudden changes. The increase in the mean temperature is associated with climatic oscillations, such as heat and cold waves [1,2,3]. Modifications of abiotic factors, such as temperature variation, can act on different biological and evolutionary aspects [4,5], may disrupt the seasonal phenology patterns of organisms, and produce unpredictable changes in ecological niches of different insect groups [6,7], affecting, for example, the tri-trophic interactions involving plants, herbivores, and parasitoids [8]. The role of temperature variation in a trophic mismatch is becoming increasingly clear, although climate changes can modify nearly every type of species interaction [6,9,10]. Anomalous and unpredictable short-term cold spells are being observed more and more frequently during the year [11] and are worth investigating in order to assess possible effects on the abundance, distribution, and function of species in a food web. Temperature variation can affect the phenology of both hosts and parasitoids [7,12,13], and asymmetric changes in the seasonal activities of closely interacting species are likely to be responsible for desynchronization in their lifecycles [8].

Insects at higher trophic levels are expected to be more strongly affected by environmental changes than organisms at lower trophic levels due to cascading effects in the food chain [14]. The parasitism rate can be affected by the phenology of parasitoid species associated with oak gall wasps, which are often multivoltine, as parasitoids must parasitize their hosts during the appropriate “window of vulnerability” [13,15]. Therefore, a variation in parasitism rates can be viewed as an immediate response to environmental factors, including intraseasonal temperature variations, climate change, and variations in habitat [13,16,17,18]. However, it is difficult to identify the factors that determine variations in the composition of a parasitoid species, and the question of why some species more successfully respond to cold stress than others remains open.

In this study, we used the Asian cynipid gall wasp (ACGW) *Dryocosmus kuriphilus* Yasumatsu and its parasitoids to investigate the response of oak gall wasps to cold stress. The ACGW is an invasive pest that affects all chestnut tree species in the genus *Castanea* (Fagales: Fagaceae) [19,20,21,22,23,24,25]. This species also represents a perturbation of the natural trophic relationships between the community of native parasitoids and oak gall wasps [26,27,28]. ACGWs were first detected in the Calabria region (southern Italy) in 2009 [29], approximately seven years after they were first detected in northern Italy (Piedmont). Heavily infested chestnut fields in the Aspromonte National Park have since been reported [18]. The ACGW is a univoltine species whose females lay eggs in chestnut buds, which induces the formation of galls on growing shoots that can inhibit shoot development and flowering [30,31]. Populations are composed entirely of females that reproduce by thelytokous parthenogenesis and can lay more than 100 eggs during their 10 days of life [32,33]. Approximately 30–40 days after oviposition, larvae from the first instar emerge and overwinter in the buds [33,34]. Larval development continues during the following spring, with two more instars developing inside the galls [35]. The authors of [36] observed that the modification of the tissues surrounding *D. kuriphilus* eggs that differentiate in order to form the larval chamber started approximately one month after oviposition. The larval chambers continued to slightly increase in size during the autumn months until January, and then grew rapidly from March to May (the period from bud swelling to bud break) [36]. Before emerging as adults between June and August, females remain inside galls in the pharate stage until ovarian maturation so that they are ready to lay eggs in new buds when they emerge [37]. Galls also differ in form (simple or compound) and the number of chambers (mono- or multichambered). Recent research on gall morphology has shown that gall mass and volume follow a pattern that may be associated with a climatic cline [38].

ACGWs have now become widespread in Europe [39], and in all invaded areas are parasitized by native parasitoids [40,41,42,43] associated with oak galls, for which the ACGW represents a non-saturated adaptation space. A large community of generalist parasitoids related to oak galls rapidly shifted to the ACGW [28,41], with local variations in the composition that depend on phenological overlaps and habitat features [13,18,44,45]. The composition of this parasitoid community depends on the morphological traits of galls (i.e., size, shape, and hardness) [30,46], on the developmental stage of the gall (i.e., young or fully formed), on the cline, and on the number of gall chambers [47]. All of these newly associated indigenous parasitoids attack concealed ACGWs through gall tissues. Larger and more fully developed galls with a higher number of chambers and thicker layers of sclerenchyma surrounding the larval chamber are negatively correlated with parasitoids. The different lengths of the ovipositors of parasitoids can, therefore, favor some species over others [30].

Other factors that affect the composition of the parasitoid community include the phenology of the host, the phenology of the parasitoids, the date of establishment of the new invasive host species, and the characteristics of the habitat [18,48]. The successful adaptation of a parasitoid to a new host requires synchrony between the presence of adult parasitoids and suitable host life stages [44].

Since 2005, the parasitoid *Torymus sinensis* Kamijo (Hymenoptera: Torymidae), a generalist and univoltine parasitoid of galling insects native to China, has been released in several European countries in order to control ACGW populations [49,50,51,52,53,54,55,56,57]. However, its introduction has altered the structure of the parasitoid community, which has recently shifted toward the ACWG [28,47,58,59]. To date, the effect of *T. sinensis* on the structure of the parasitoid community in oak galls has been minimal [60].

Considering the increase in the frequency of extreme climatic events over the last twenty years (e.g., low temperatures during the vegetative period of the chestnut tree; see Figures S1 and S2), this study aimed to elucidate the potential effect of cold stress on both ACGW biology and parasitism by native and introduced parasitoids.

The ACGW–parasitoid system represents an ideal subject in which to evaluate the effect of sudden extreme climatic events due to the wasps’ biological characteristics, which include the ability to complete development in galls detached from plants. The obtained results may guide further experimental studies on the effects of sudden and extreme temperature events on oak gall wasps.

## 2. Materials and Methods

### 2.1. Study Area

Shoots with galls from three chestnut fields were collected weekly between May and July in 2017 and 2018. The chestnut trees are located on a plateau within the Aspromonte Mountains (Reggio Calabria, southern Italy) (latitude 38°3′, longitude 15°44′, 980 m above sea level (asl)). The fields are adjacent to farmland on which other fruit crops (e.g., cherries and apples) and vegetable crops (e.g., cereals and potatoes) are grown. Downy oak (*Quercus pubescens* Willd), European oak (*Quercus robur* L., etc.), and holm oak (*Quercus ilex* L.) are widespread throughout the study area. The chestnut fields include two orchards (with 25- and 50-year-old trees, respectively) and one coppice (with 15-year-old trees), which are denoted A, B, and C, respectively. The chestnut orchards are made up of wild plants grafted with the local Nzerta cultivar, while the coppice is made up of trees obtained from seedlings of the same cultivar. Fields are managed without pesticides, growth regulators, or fertilizers. *Torymus sinensis* has been introduced into the study area (3–4 km away), but is not used in the experimental fields.

### 2.2. Study Design

The experiment’s design considered both the natural parasitization by different parasitoid species of ACGW and the effects of experimentally induced cold stress on parasitization and ACGW emergence. Sampling was performed weekly. The cold stress tests were repeated during the spring–summer period to ensure that all parasitoid species involved in the biological control of ACGWs were present.

### 2.3. Field Sampling and Seasonal Parasitism

We adopted an observational study design with sequential sampling throughout the larva-to-adult life stage. Shoots with galls from fields A, B, and C were collected from May until adult ACGWs emerged, which occurs in the study area in July. Approximately 24 shoots were collected each week from each chestnut field (A, B, and C), with one shoot collected in each ordinal direction (NE, SE, SW, and NW) from 6 trees. Thirty-six galls (12 for each chestnut field) were randomly chosen from the shoots (nine galls per ordinal direction) and carefully dissected under a stereomicroscope (SZX9, Olympus^®^, Tokyo, Japan) within 24 h of collection. All chambers and vital and non-vital stages of gall wasps and parasitoids (i.e., larvae, pupae, and pharate adults) were recorded. The parasitism rate was calculated as the number of chambers with living parasitoids (larvae, pupae, adults, emerged insects) divided by the total number of chambers, excluding chambers damaged by rot and without an identifiable host.

### 2.4. Cold Stress Experiment

To evaluate the effect of cold stress on the composition of parasitoid species, 30 galls (hereafter referred to as non-thermally treated, NTT) were chosen each week from the collected shoots, individually placed in closed alveolar containers containing cotton, and stored at room temperature (ranging from 14 to 25 °C). Simultaneously, the cut surfaces of selected shoots (15 per field) were coated with wax and the leaves were removed to prevent them from wilting. Each shoot was wrapped in plastic film and stored at a controlled temperature (8 °C) in a laboratory fridge for 7 days. This temperature is lower than the theoretical value at which ACGWs stop developing but do not die [13]. The length of the interval was chosen on the basis of preliminary tests that showed that too short an interval would have produced less evaluable effects while too long an interval would have irreparably damaged the biological material, and why a 7-day interval is more manageable.

The shoots were then repositioned on trees in the field where they were collected in order to evaluate the response of ACGWs and parasitoids to cold stress. Specifically, the shoots were tied to the branches of chestnut trees in each field (approximately five shoots per plant) using plastic-annealed iron wires. After one week, the shoots were collected and carried back to the laboratory. Thirty cold-stressed (TT) galls were isolated in alveolar containers to allow for the emergence of ACGW adults and/or parasitoids. The TT galls were kept in a walk-in laboratory room at temperatures ranging from 14 to 25 °C. Each gall was inspected once per week during the May–September period and every two weeks during the October–April period.

### 2.5. Emergence of the ACGWs and Parasitoids

All individuals that emerged from NTT and TT galls were separated, identified, and stored in alcohol. Identification was conducted using the taxonomic keys [27,61,62,63,64,65]. Specimens were then compared with individuals identified by the Institute for Sustainable Plant Protection based on molecular protocols (COI, 28S, and ITS2) following [66,67].

Due to the difficulty of separating out the cryptic species present in the *Torymus flavipes* Walker complex and species of the genus *Eupelmus* [65,68], some analyses were performed on the basis of aggregated data at the genus level for *Eupelmus* spp. and at the morphospecies level for *T. flavipes*. Voucher specimens were deposited at the LEEA Laboratory (Laboratorio di Entomologia ed Ecologia Applicata), Università Mediterranea di Reggio Calabria, Italy.

### 2.6. Historical Climate Data

To identify the frequency of the intervals during which cold stress acted on the ACGW and its parasitoids, we collected from the Arpacal regional environmental monitoring station 20 years (2000–2019) of historical data on the temperature in the same area and at the same altitude as the studied fields.

### 2.7. Statistical Analysis

Data on the number of chambers in galls were tested for normality using Kolmogorov–Smirnov (K-S) tests (*p* = 0.05). The number of chambers in dissected galls in each year was compared using analysis of variance (ANOVA). The data were log-transformed to meet the assumption of normality. The model included as variables chestnut fields (*n* = 3; A, B, or C), type of gall (*n* = 2; simple or compound, sensu [33], ordinal direction (*n* = 4; NE, SE, SW, or NW), and interaction between chestnut fields and type of gall. We used a generalized linear model of binomial data (0 = chambers not parasitized; 1 = chambers parasitized) to compare differences in the parasitism rates of dissected galls collected each year. The following categorical variables were included in the model: chestnut field (A, B, or C), ordinal direction (NE, SE, SW, or NW), and gall type (simple or compound). Lastly, the date of collection was included as a continuous variable. We restricted our analysis to the distinguishable life stages of gall wasps and their parasitoids.

To perform an overall evaluation of the cold stress effects that went beyond the variability linked to other variables (coppice or orchard and different neighboring vegetation), the data were pooled across chestnut fields, and the number of ACGWs and parasitoids that emerged from NTT and TT galls was compared using a chi-squared (χ^2^) test of the equality of distributions in each year. For species in the genus *Eupelmus*, another chi-squared (χ^2^) test was used to assess the differences between species in the different treatment groups. The -2Log Likelihood value was used to assess the distribution of emergent adults according to the sampling date of the TT and NTT treatments. This latter test was performed only for the most significant species that emerged in this study.

The historical climatic data for the twenty years (2000–2019) from the monitoring station (*n*° 2465) were used to determine the frequency of short periods of time (at least 4 days) during which the minimum daily temperature was less than or equal to 8.5 °C.

Historical temperature variations in the cold periods from April to August over the twenty years were analyzed using the linear regression method.

We used SPSS v.23 [69] for all data analyses and [70] to produce graphs. All data are expressed as untransformed mean values ± the standard error (SE).

## 3. Results

### 3.1. Field Sampling and Seasonal Parasitism

We found a total of 7423 chambers in the galls (3980 in 2017 and 3443 in 2018). The K-S test values were 0.135 in 2017 (*p* < 0.001) and 0.139 in 2018 (*p* < 0.001). The number of chambers per simple gall ranged between 1 and 18 in 2017 and 1 and 24 in 2018. The number of chambers per compound gall ranged between 2 and 37 in 2017 and 2 and 28 in 2018. The mean number of chambers was significantly higher in compound galls than in simple galls in both years (2017: simple = 5.73 ± 0.25, *n* = 174; compound = 11.71 ± 0.37, *n* = 256; 2018: simple = 6.03 ± 0.28, *n* = 238; compound = 10.68 ± 0.39, *n* = 189) (Table 1). Therefore, in the second year of the monitoring period, the number of chambers in simple galls increased, while that in compound galls decreased.

**Figure 1:**
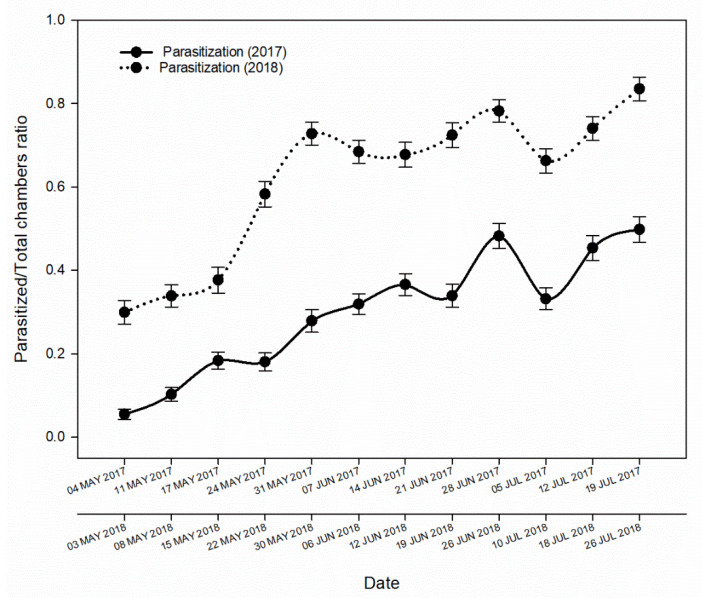
The proportion of chambers parasitized during the monitoring periods in 2017 and 2018. The data collected each year in the three chestnut fields were pooled.

The ACGW parasitism rate was affected by several variables (Table 2). The parasitism rate varied between chestnut fields and sampling dates in each year but not between ordinal directions or gall typologies (Table 2). The lowest parasitism rate was recorded during 2017 in all monitored fields (Table 3), with about half of all hosts parasitized, whereas the ratio was ≃ 0.8 in 2018 (Figure 1).

The parasitism rate was significantly higher in field C (the coppice chestnut field) than in fields A and B (the orchard chestnut fields) in both years (Table 3). The parasitism rates also differed between sampling time points (2017: first week = 0.055, last week = 0.498; 2018: first week = 0.299, last week = 0.835) (Figure 1).

### 3.2. Cold Stress Experiment

**Figure 2:**
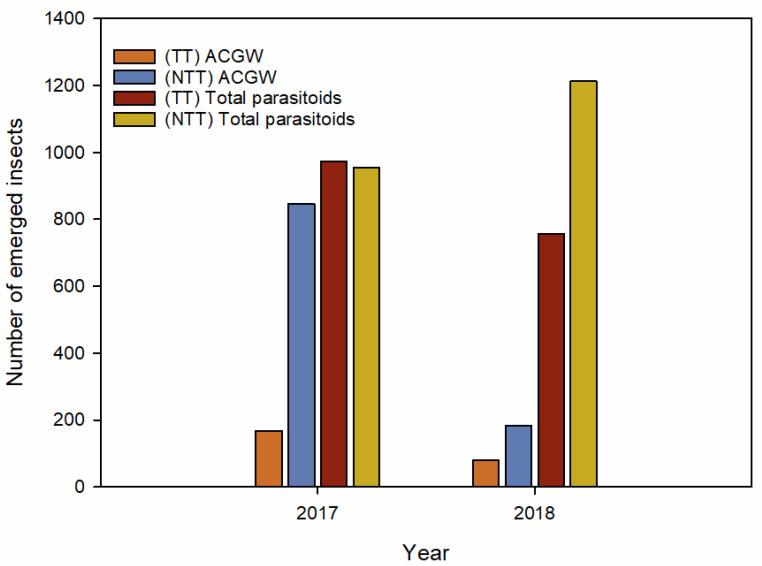
Bar charts depicting the number of Asian cynipid gall wasps (ACGWs) and parasitoids in cold-stressed (TT) galls and non-cold-stressed (NTT) galls collected in different years from different fields. For experimental details, see the text. Data from the three examined chestnut fields were pooled.

The total number of emergent ACGWs and parasitoids differed between years, with a larger number of individuals collected in 2017 compared with 2018 (2941 vs. 2230). The chi-square tests revealed significant differences among the adults (ACGW and parasitoid) that emerged from the TT and NTT treatment groups in both years (2017: χ^2^_(17, 2941)_ = 598, *p* < 0.001; 2018: χ^2^_(16, 2230)_ = 234, *p* < 0.001). The number of ACGW adults that emerged was lower in the TT treatment group than in the NTT treatment group (2017: NTT = 845, TT = 168; 2018: NTT = 182, TT = 80) (Figure 2). In 2017, the number of parasitoids in each of the two groups was similar (NTT = 984, TT = 974), but differed between groups in 2018 (NTT = 1213, TT = 755) (Figure 2).

### 3.3. Emergence of the ACGW and Its Parasitoids

**Figure 3:**
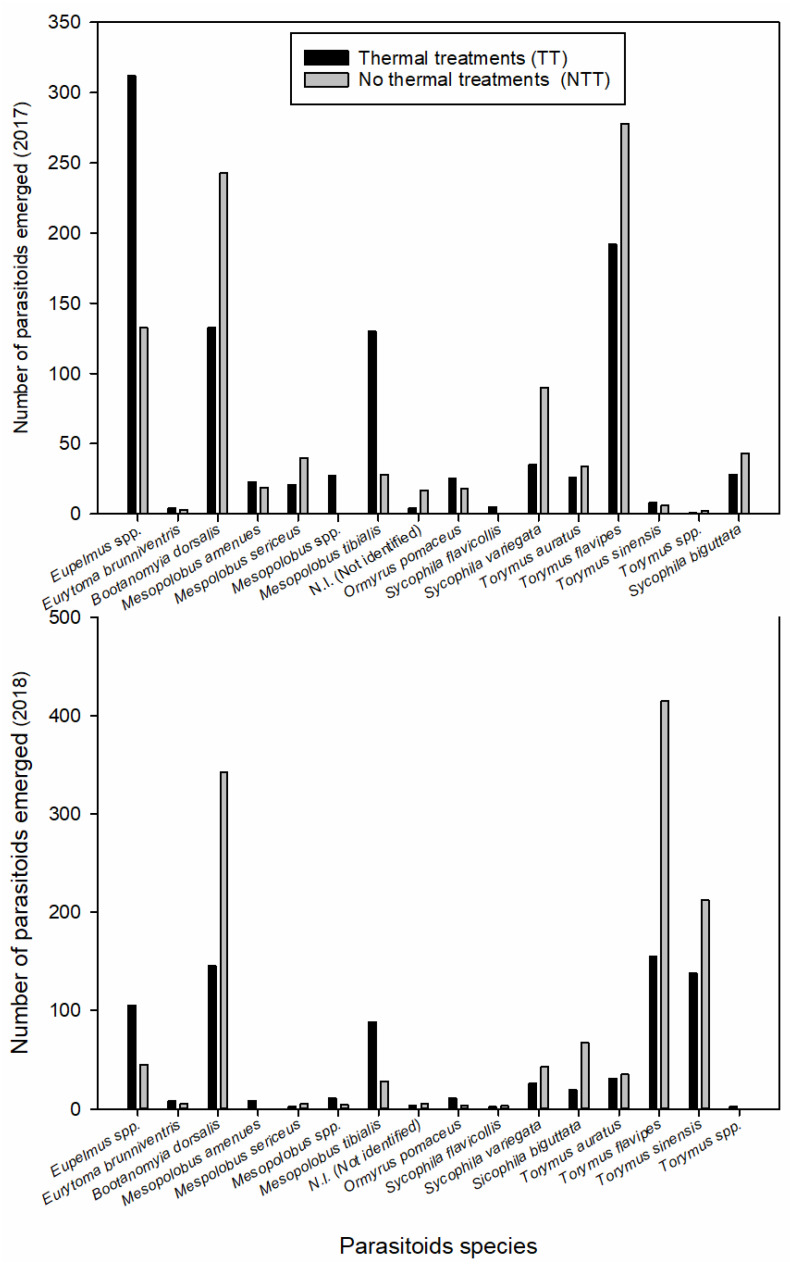
Parasitoid species that emerged from galls in different treatment groups from the three monitored chestnut fields. The data from the three fields were pooled by year.

**Figure 4:**
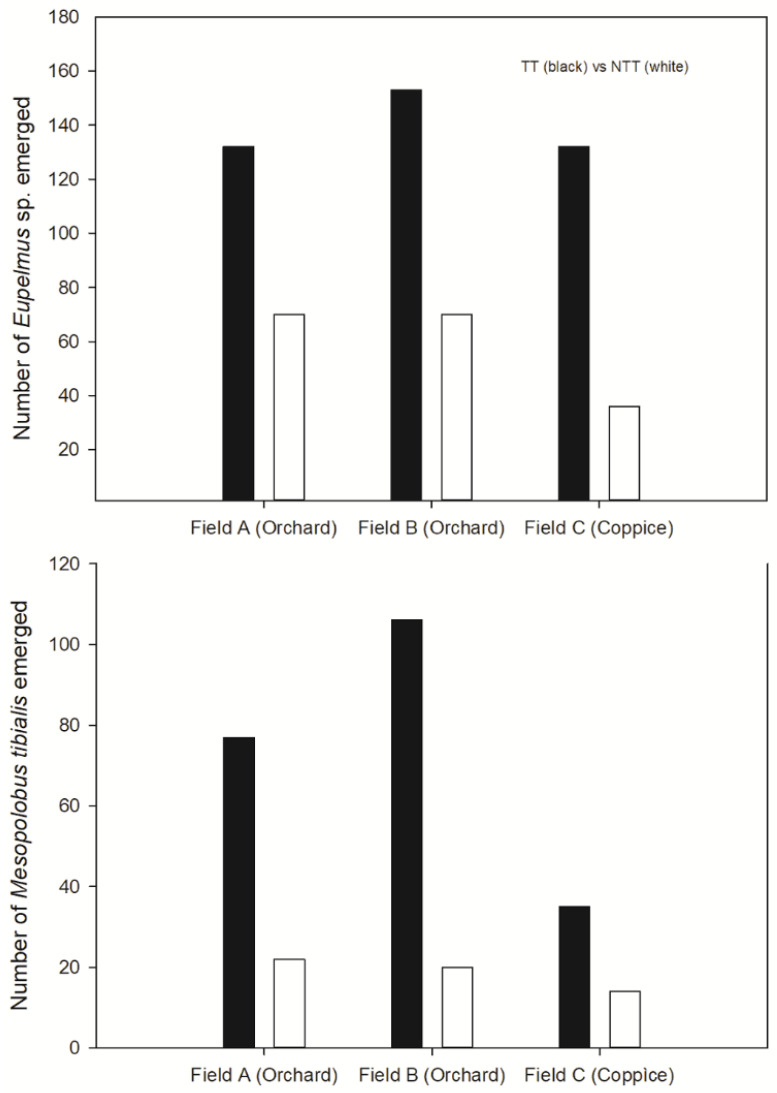
*Eupelmus* spp. and *Mesopolobus tibialis* emerged from cold-stressed (TT) and non-cold-stressed (NTT) galls collected from three chestnut fields. Data from the two years were pooled.

A decrease in the number of parasitoids that emerged from TT galls was found for the following species: *Bootanomyia dorsalis* (F), *T. flavipes*, *Sycophila biguttata* (Swederus), and *Sycophila variegata* (Curtis, 1831) (Figure 3). In contrast, the number of emergent specimens belonging to the genus *Eupelmus* and *Mesopolobus tibialis* (Westwood) was 2-fold higher in the TT treatment group than in the NTT treatment group (Figure 4), whereas the number of *Torymus auratus* (Geoffroy in Fourcroy, 1785) in each of the two treatment groups was similar (Figure 3). *Eupelmus* spp. specimens were the most abundant in all chestnut fields. Three species belonging to the genus *Eupelmus* were detected, although they were found to have different relative frequencies. The most abundant species was *Eupelmus azureus* Ratzeburg (76.4%), followed by *Eupelmus urozonus* Dalman (21.1%) and *Eupelmus kiefferi* De Stefani (2.5%). The *X*^2^ tests revealed significant differences in *Eupelmus* spp. between treatment groups only in 2017 (2017: χ^2^_(2, 433)_ = 23.543, *p* <0.001; 2018: χ^2^_(2, 160)_ = 2.91, *p* = 0.256) and between chestnut fields in both years (2017: χ^2^_(4, 433)_ = 14.87, *p* = 0.005; 2018: χ^2^_(4, 160)_ = 9.64, *p* = 0.047) (Figure S3). The number of observed *E. azureus* adults was higher than expected in the TT treatment group (316 vs. 299) and lower than expected in the NTT treatment group (137 vs. 154). The comparison between the distributions of adults that emerged in relation to the monitoring date highlighted a difference between the TT and NTT treatment groups (*p* < 0.001) (Figure S4).

Both the increase in the frequency of *Eupelmus* spp. and *M. tibialis* and the decrease in the frequency of such species as *B. dorsalis* were observed during the entire sampling period.

### 3.4. Historical Climate Data

The historical temperature data on the study area showed that the time intervals of at least 4 consecutive days with a minimum daily temperature of 8.5 °C or less mainly occurred between May and June (69 and 11 intervals) (Figure S1). In the analyzed 20-year period, only shorter intervals (1–3 days) were detected in July at temperatures close to 8.5 °C. Time intervals with minimum daily temperatures lower than or equal to 8.5 °C for at least 4 consecutive days became increasingly frequent in May (R square = 0.569; df = 2.18; *p* < 0.001; Figure S2).

## 4. Discussion

In our experimental approach, we simulated cold stress events, which caused a variation in the ratio of parasitoid species. The analysis of the temperatures in our study area over the past twenty years indicates that cold snaps now occur more frequently in May. Unpredictable cold waves have a direct and more easily verifiable effect on plants. The authors of [71] highlight that, despite the increase in the mean annual temperature, unpredictable cold waves may retard the expansion of plants to higher altitudes and latitudes.

We have shown that ACGW parasitism and mortality in the three chestnut fields were affected by the cold stress. The ACGW population was smaller in 2018 than in 2017, and this was found to be associated with higher parasitism rates in galls. The parasitism rate varied in single seasons (from May to July), and different mean parasitism rates were found between years and between chestnut fields. In particular, higher parasitism rates were found in the coppice field, which is consistent with previous results from studies in the same chestnut fields (2013–2015) [18].

This rate of proliferation is a clear indication of the adaptation of this species to the ACGW in the study area and confirms results recorded in Italy and other European countries [54,56,57,72,73,74].

Cold stress did not affect the composition of the parasitoid community in any chestnut field, which is consistent with the findings reported in [75], where no differences were found in the diversity, richness, or evenness of ACGW-associated species despite differences in their habitat. Conversely, [76] showed that native parasitoid communities that emerged from galls differed between pure and mixed chestnut stands at the same altitude, even though there was no difference in their abundance. A regulatory and compensatory effect in the context of overall parasitization with a reduction in the presence of some species and an increase in the presence of other species was highlighted in this study. Our results reveal species-specific differences in the abundance and performance of parasitoids associated with ACGW in response to cold stress. In particular, the frequency of *Eupelmus* spp. and *M. tibialis* doubled in the cold treatment group in all three chestnut fields in both years. Typically, *M. tibialis* parasitizes the ACGW in early May (see Figure S4) and completes its development quickly. In contrast, *Eupelmus* spp. females are active later in June and usually the last parasitoids to emerge.

The high proportion of specimens belonging to the genus *Eupelmus* recorded at all TT sample sites seems be related to the greater presence of *Eupelmus azureus* because, despite the fact that three species of *Eupelmus* were detected in all fields, the frequency of *E. azureus* increased after the cold stress treatment, while *E. urozonus* and *E. kiefferi* were less affected.

The increase in *E. azureus* may be related to the greater ability of this species to develop as a hyper-parasitoid [20,77], decaying hosts (a reduction in the emergence and therefore an increase in ACGW mortality was observed in 2018), or to its higher resistance to cold stress.

However, the greater presence of *E. azureus* could also be linked to variations in emissions of herbivore-induced plant volatiles in response to cold stress that might be more attractive to this species [78].

Cold stress, although limited to a one-week period, prolonged the development of the juvenile stages of the ACGW and its parasitoids inside galls and delayed the emergence of parasitoids. This prolonged phenology may have produced a wider temporal window for parasitism by some parasitoid species. Furthermore, the re-exposure of galls in trees to cold stress could have caused excessive drying and possibly resulted in the emission of plant volatile substances attractive to *Eupelmus* spp. and *M. tibialis*. The variations in the water content of the galls could also have affected the parasitoids’ success.

Cold stress was found to negatively affect the abundance of *T. flavipes, T. sinensis*, *S. biguttata*, and *Ormyrus pomaceus* (Geoffroy). Interestingly, all of these species can develop via the same larval stages as *M. tibialis*. Furthermore, our results confirm that some parasitoids, such as *Torymus auratus* (Geoffroy in Fourcroy, 1785) and *B.* *dorsalis*, prefer to attack during the formation of pupae. These parasitoid species, along with *S. variegata* (Curtis), were the last to emerge during the final few days of June and during July [42]. In addition, their abundance was also reduced by cold stress.

## 5. Conclusions

This study improves our understanding of the effects of cold stress on insect growth and the succession of host life stages, and provides information on parasitoids’ adaptation to new host phenologies. A new host can share parasitoids that exist in the area, but the processes that influence their adaptation are far more complex than the simultaneous presence of the adult parasitoid and the susceptible stage of the host. Therefore, this study focused on variations that occur in the parasitoid community due to simple phenological variations and stress conditions. We have shown that exposure of galls to low temperatures affects the structure of the parasitoid community. Understanding which processes affect the structure of a parasitoid community is fundamental, particularly when a phytophagous insect shifts toward a new host, as has occurred with the ACGW and *C. sativa*.

## Figures and Tables

**Table 1:** Analysis of variance (ANOVA) evaluating the effect of different variables and their interaction on the number of chambers in galls (Levene’s test; 2017: F = 1.19; df = 23, 405; *p* = 0.25; 2018: F = 1.12; df = 23, 404; *p* = 0.32).

	Source	*Df*	*FI*	*p*
2017	Intercept	1	5135.96	<0.001
	Gall type (Simple, Compound)	1	172.26	<0.001
	Chestnut field	2	13.55	<0.001
	Ordinal direction	3	0.89	0.45
	Gall type * chestnut field	2	6.30	0.02
2018	Intercept	1	3531.15	<0.001
	Gall type (Simple, Compound)	1	119.92	<0.001
	Chestnut field	2	2.06	0.13
	Ordinal direction	3	0.48	0.70
	Gall type * chestnut field	2	0.885	0.413

The asterisk * is in common use to indicate interactions among the variables that it joins.

**Table 2:** The binomial generalized linear model (GLM)’s evaluation of the effects of different variables on the parasitism rate (2017: *n* = 3784; 2018: *n* = 3053).

	Source	*Df*	Wald Chi-Square	*p*
2017	Intercept	1	296.93	<0.001
	Gall type (Simple, Compound)	1	0.88	0.349
	Chestnut field	2	162.75	<0.001
	Date	1	296.78	<0.001
	Collection site	4	2.49	0.647
2018	Intercept	1	244.93	<0.001
	Gall type (Simple, Compound)	1	0.87	0.350
	Chestnut Field	2	21.68	<0.001
	Date	1	244.98	<0.001
	Collection site	3	4.22	0.239

**Table 3:** Parasitism rate (the number of chambers with living parasitoids (larvae, pupae, adults, emerged insects) divided by the total number of chambers, excluding chambers damaged by rot and without an identifiable host) in the chestnut fields during the monitoring period. *n* = total number of chambers.

Year				
Chestnut Field	2017		2018	
		*n*		*n*
A	0.18	1435	0.57	1031
B	0.33	1348	0.59	1048
C	0.40	1001	0.67	974

## Supplementary Materials

The following are available online at https://www.mdpi.com/2075-4450/11/12/841/s1, Figure S1: Relationship among monitoring months and interval time with daily minimum temperature lower than or equal to 8.5 degrees for 4 consecutive days (the data refer to twenty years, 2000–2019). Historical data were collected from the meteorological station that monitored the study area. Figure S2: Relationship among years (May and June) and interval time with daily minimum temperature lower than or equal to 8.5 °C for at least 4 consecutive days (the data refer to twenty years, 2000–2019). Historical data were collected from the meteorological station that monitored the study area (n° 2465). Regression line on May (f = y0 + a*x; R square = 0.569; df = 2, 18; *p* < 0.001). Figure S3: Relationship among *Eupelmus* species at different chestnut fields (two orchards and one coppice) (left) and at different treatments (NTT = no temperature treatment, TT = temperature treatment). Figure S4: Relationships among the date of monitoring of the galls and some emergent parasitoids. *Eupelmus* sp (2017: log.Lik_12,443_ = 173.44, *p* < 0.001; 2018: log.Lik_11,150_ = 99.52, *p* < 0.001), *Mesopolobus tibialis* (2017: log.Lik_9158_ = 132.10, *p* < 0.001; 2018: log.Lik_5116_ = 37.04, *p* < 0.001), *Bootanomyia dorsalis* (2017: log.Lik_9376_ = 61.10, *p* < 0.001; 2018: log.Lik_10,489_ = 105.68, *p* < 0.001) and *Torymus flavipes* (2017: log.Lik_9158_ = 124.73, *p* < 0.001; 2018: log.Lik_5116_ = 37.10, *p* < 0.001) are compared by way of example (NTT = no temperature treatment, TT = temperature treatment).
