# Supplementary material for: Short-Term Cold Stress Affects Parasitism on the Asian Chestnut Gall Wasp Dryocosmus kuriphilus
Source: Insects. 2020 Nov 28;11(12):841. doi: 10.3390/insects11120841 (PMC7760994; doi:10.3390/insects11120841)
Supplement: Supplementary file 1 [file insects-11-00841-s001.pdf]

Data Supplemental to "Short-Term Cold Stress Affects Parasitism on the Asian Chestnut Gall Wasp *Dryocosmus kuriphilus*"

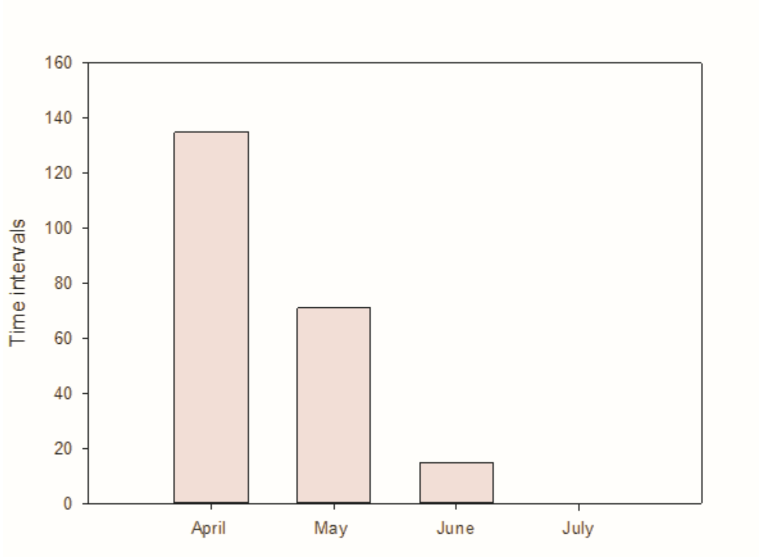

Figure S1. Relationship among monitoring months and interval time with daily minimum temperature lower than or equal to 8.5 degrees for 4 consecutive days (the data refer to twenty years, 2000–2019). Historical data were collected from the meteorological station that monitored the study area.

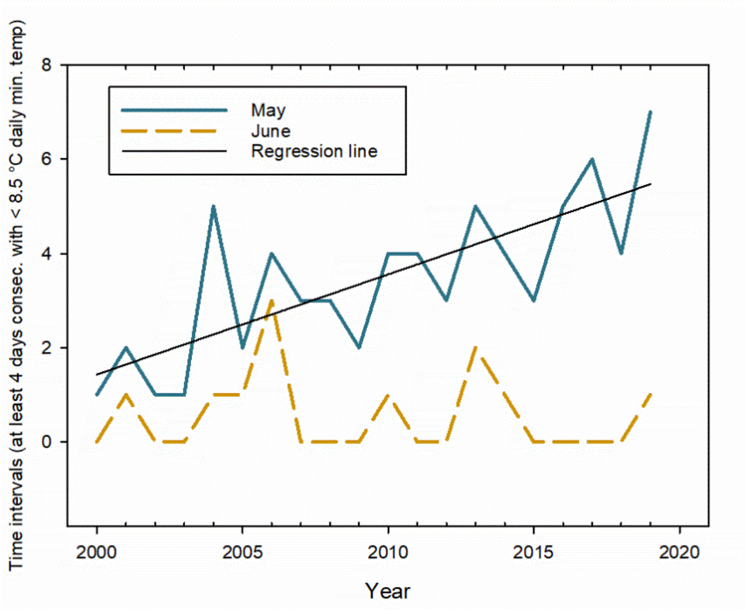

Figure S2. Relationship among years (May and June) and interval time with daily minimum temperature lower than or equal to 8.5 °C for at least 4 consecutive days (the data refer to twenty years, 2000–2019). Historical data were collected from the meteorological station that monitored the study area (n° 2465). Regression line on May (f = y0 + a*x; R square = 0.569; df = 2, 18; $p < 0.001$).

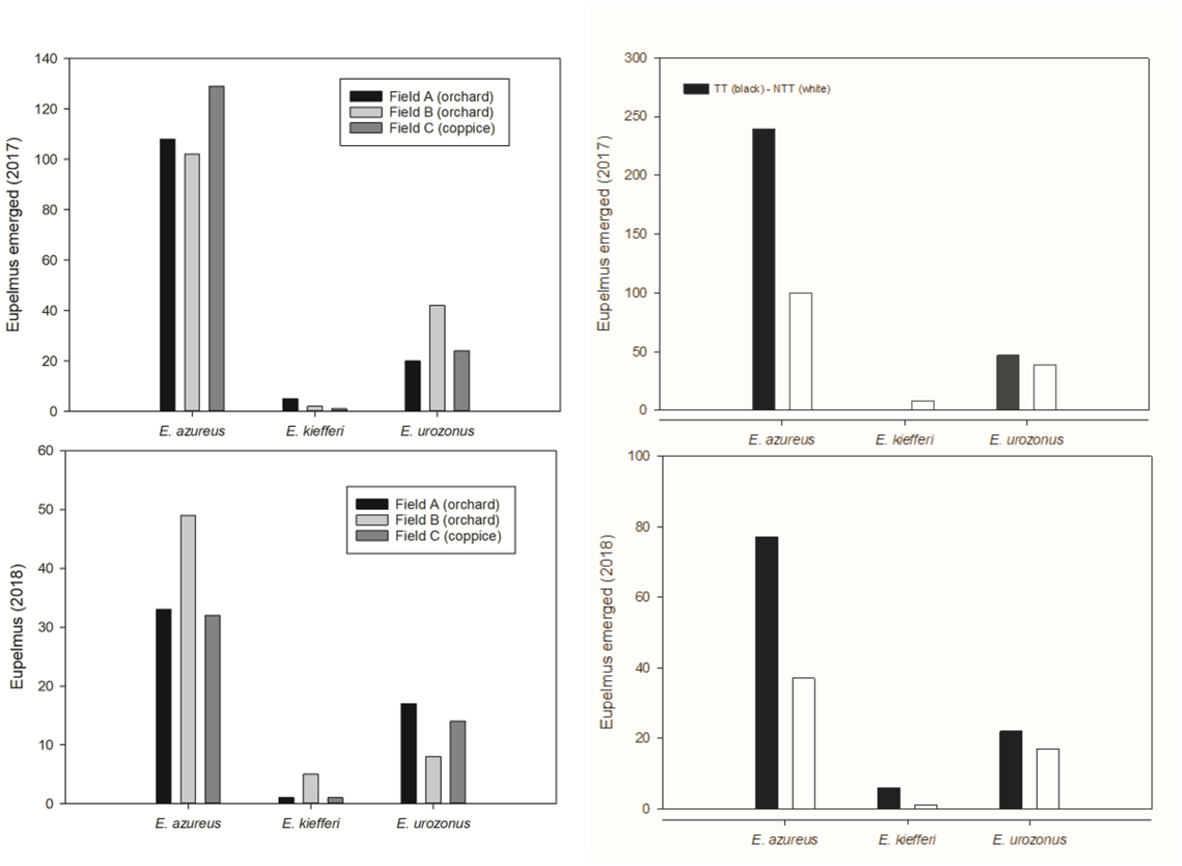

Figure S3. Relationship among *Eupelmus* species at different chestnut fields (two orchards and one coppice) (left) and at different treatments (NTT = no temperature treatment, TT = temperature treatment).

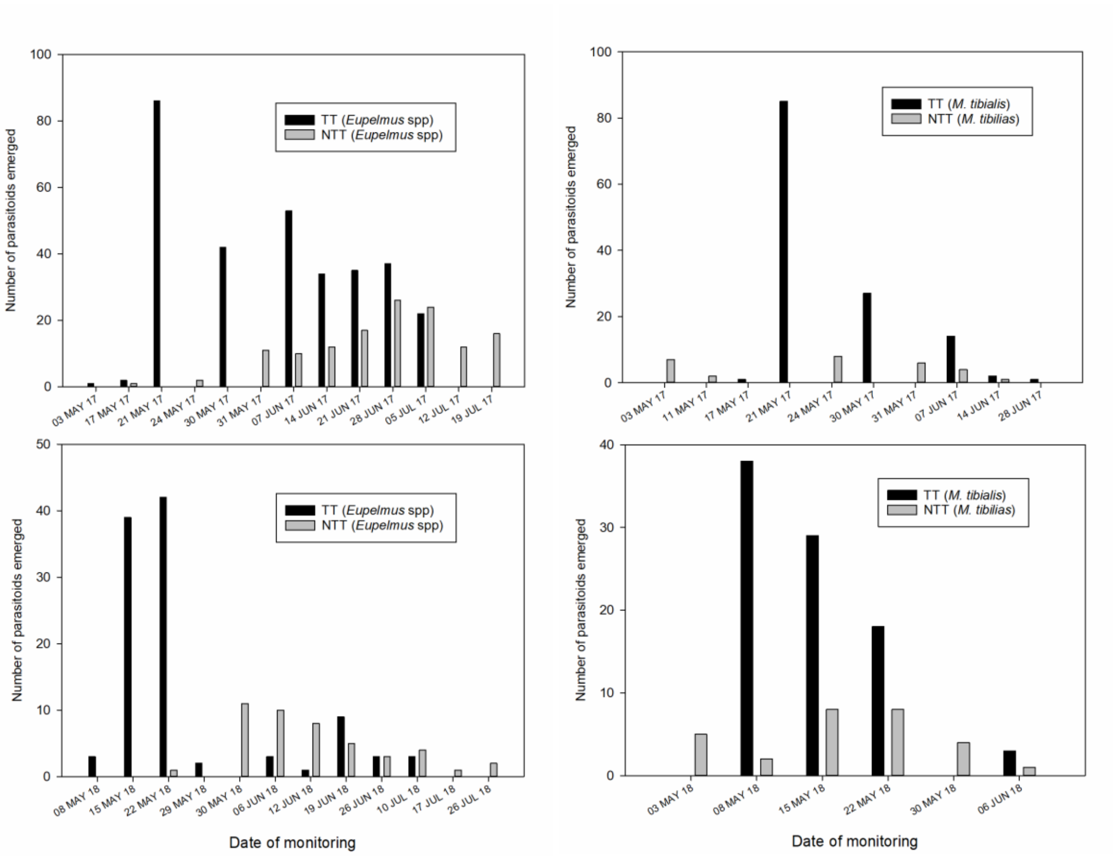
Number of parasitoids emerged
TT (Eupelmus spp)
NTT (Eupelmus spp)
03 MAY 17
17 MAY 17
21 MAY 17
24 MAY 17
30 MAY 17
31 MAY 17
07 JUN 17
14 JUN 17
21 JUN 17
28 JUN 17
05 JUL 17
12 JUL 17
19 JUL 17
TT (M. tibialis)
NTT (M. tibilias)
03 MAY 17
11 MAY 17
17 MAY 17
21 MAY 17
24 MAY 17
30 MAY 17
31 MAY 17
07 JUN 17
14 JUN 17
28 JUN 17
08 MAY 18
15 MAY 18
22 MAY 18
29 MAY 18
30 MAY 18
06 JUN 18
12 JUN 18
19 JUN 18
26 JUN 18
10 JUL 18
17 JUL 18
26 JUL 18
03 MAY 18
Date of monitoring

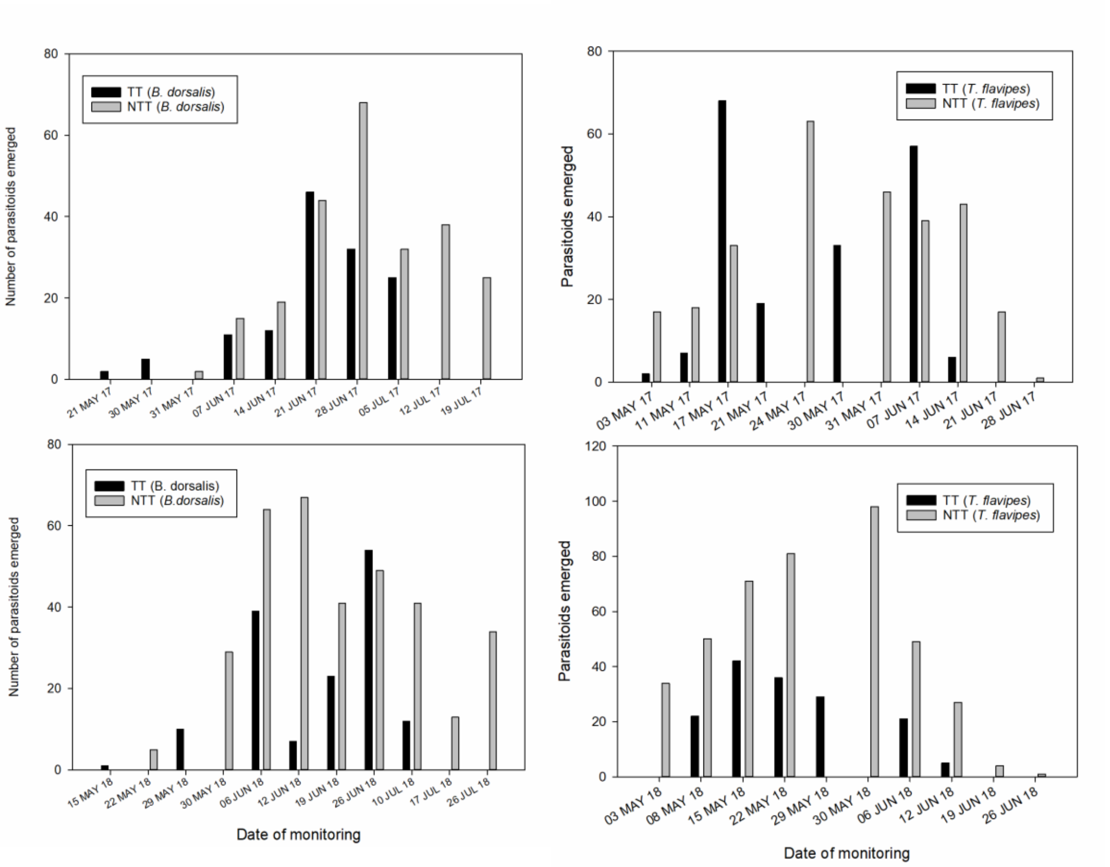
Number of parasitoids emerged
TT (B. dorsalis)
NTT (B. dorsalis)
21 MAY 17
30 MAY 17
31 MAY 17
07 JUN 17
14 JUN 17
21 JUN 17
28 JUN 17
05 JUL 17
12 JUL 17
19 JUL 17
Parasitoids emerged
TT (T. flavipes)
NTT (T. flavipes)
03 MAY 17
11 MAY 17
17 MAY 17
21 MAY 17
24 MAY 17
30 MAY 17
31 MAY 17
07 JUN 17
14 JUN 17
21 JUN 17
28 JUN 17
15 MAY 18
22 MAY 18
29 MAY 18
30 MAY 18
06 JUN 18
12 JUN 18
19 JUN 18
26 JUN 18
10 JUL 18
17 JUL 18
26 JUL 18
03 MAY 18
08 MAY 18
Date of monitoring

Figure S4. Relationships among the date of monitoring of the galls and some emergent parasitoids. *Eupelmus* sp (2017: $\text{log.Lik}_{12,443} = 173.44$, $p < 0.001$; 2018: $\text{log.Lik}_{11,150} = 99.52$, $p < 0.001$), *Mesopolobus tibialis* (2017: $\text{log.Lik}_{9158} = 132.10$, $p < 0.001$; 2018: $\text{log.Lik}_{5116} = 37.04$, $p < 0.001$), *Bootanomyia dorsalis* (2017: $\text{log.Lik}_{9376} = 61.10$, $p < 0.001$; 2018: $\text{log.Lik}_{10,489} = 105.68$, $p < 0.001$) and *Torymus flavipes* (2017: $\text{log.Lik}_{9158} = 124.73$, $p < 0.001$; 2018: $\text{log.Lik}_{5116} = 37.10$, $p < 0.001$) are compared by way of example (NTT = no temperature treatment, TT = temperature treatment).
